# Case of Incontinence of Urine, Cured by Tinctura Lyttæ

**Published:** 1819-03

**Authors:** John Maclean

**Affiliations:** Edinburgh.


*For the London Medical and Physical Journal.*

### *Case of Incontinence of Urine, cured by Tinctura Lyttæ;* by JOHN MACLEAN, M.D. Edinburgh.

HUGH MITCHEL, a brewer's drayman, aged 42, had been addicted for several years to excess in spirituous liquors, and ultimately became affected with incontinence of urine; which had continued for a year when he applied to me for advice, in a state of great distress from the disease, and despondency of recovery.

Attributing the complaint to relaxation of the urinary passages from habitual intemperance, I directed him to abstain entirely from spirituous liquors, to live chiefly on animal food, and to use cinchona, sea-bathing, &c. with a view to restore the tone of the debilitated organs. This plan, however, having been strictly pursued for a long time with little advantage, I prescribed the tinct. lyttæ in small doses, gradually increased to twenty drops three times a-day, combined with demulcent drinks. In a few weeks slight strangury supervened, but subsided on discontinuing the tincture; and shortly after, by perseverance in the regimen directed, and the occasional use of the cantharides, he became entirely free from the incontinence of urine; and, during a considerable interval which has since elapsed, has had no return of the complaint.

*Edinburgh; Nov.* 6, 1818.

